# Die etimologie en verstaan van rampe deur die lens van Afrikaans as moedertaal

**DOI:** 10.4102/jamba.v17i1.1899

**Published:** 2025-04-16

**Authors:** Gideon Wentink, Johanita Kirsten, Leandri Kruger, Christo Coetzee

**Affiliations:** 1 Unit for Environmental Sciences and Management, Faculty of Natural and Agricultural Science, North-West University, Potchefstroom, South Africa; 2 Research Focus of Understanding and Processing Language in Complex Settings, Faculty of Humanities, North-West University, Vanderbijlpark, South Africa

**Keywords:** disaster, crises, catastrophe, disaster risk reduction, Afrikaans, mother tongue, etymology

## Abstract

**The etymology and understanding of disasters through the lens of Afrikaans as a mother tongue.** The historical development of Afrikaans had a modest yet complex beginning. Over time, Afrikaans has evolved into a fully developed scientific language in various contexts, including in disaster studies. To enhance our understanding of disasters and the natural environment, several disaster-related terms have emerged in Afrikaans, namely ‘disaster’, ‘crisis’, and ‘catastrophe’. This study therefore aimed to conduct an in-depth analysis of the unique disaster-related terminology used in Afrikaans and to explore what this terminology reveals about Afrikaans speakers’ conceptualisation of disasters. A qualitative research approach was followed, employing in-depth Corpus and N-gram analyses of various disaster-related Afrikaans terms. The findings indicate that while Afrikaans speakers differentiate in their use of the terms ‘disaster’, ‘crisis’, and ‘catastrophe’, the core meanings of these terms remain consistent. Although the analyses revealed no explicit focus on disaster risk reduction, it did show that a humanitarian component underpins these terms. This suggests that their usage aligns with their function within the scientific language.

**Contribution:**

Afrikaans, as a mother tongue, thus plays a fundamental role in shaping the etymology and understanding of disasters, as well as in advancing sustainable disaster risk reduction and disaster management practices.

## Inleiding

Die Afrikaanse taal het sy oorsprong met die aankoms van Nederlandse setlaars aan die Kaap die Goeie Hoop in April 1652 om namens die Vereenigde Oostindische Compagnie (VOC) ’n halfwegstasie op te rig vir skepe op pad na Indië en Suidoos-Asië (Dye & La Croix 2020:33; Oliver 2019:2). As gevolg van die multikulturele konteks en die historiese dryfvere wat in die vroeë koloniale Suid-Afrika van toepassing was, het Afrikaans na vore getree as ’n Kreoolse taal met invloede uit Nederlands, Maleis, Frans, Duits en verskeie inheemse tale wat destyds in die streek gepraat is (Groenewald 2019:10). By sy ontstaan, is Afrikaans dikwels as ‘kombuishollands’ bespot – ’n verwysing na die feit dat dit hoofsaaklik deur burgers van laer sosio-ekonomiese klasse en slawe gepraat is (Hamans 2021:26). Sedert hierdie nederige begin het Afrikaans egter ontwikkel tot ’n volwaardige wetenskapstaal wat in verskeie lande wêreldwyd gebruik word (Alberts 2013:6).

Suid-Afrika is ’n land wat gereeld deur droogtes, vloede en siekte-uitbrekings onder mense en diere geraak word, wat dan dikwels verwoestende verliese aan lewens en lewensbestaan tot gevolg het (Meza et al. 2021:2; Sirdar et al. 2021; Twumasi 2017). Gevolglik het Afrikaans, as deel van sy ontwikkeling as wetenskapstaal, ook gepoog om ’n beter begrip van Suid-Afrika se natuurlike omgewing en sy verskeie rampe te fasiliteer. In hierdie konteks het verskeie iterasies van woorde soos ‘ramp’, ‘krisis’ en ‘katastrofe’ in Afrikaans na vore gekom om soortgelyke, maar terselfdertyd unieke konsepte en perspektiewe op rampgebeure te verduidelik wanneer dit manifesteer. Hierdie artikel poog om uit te brei oor unieke woorde wat in Afrikaans gebruik word vir rampverwante terminologie en wat hierdie terminologie oor Afrikaanssprekendes se begrip van rampe onthul.

Om hierdie doel te bereik, word daar eers gekyk na die ontwikkeling van Afrikaans as wetenskapstaal. Daarbenewens bespreek die artikel die belangrike rol wat die ontwikkeling van rampverwante terminologie in tale soos Afrikaans speel – nie net om ’n begrip van rampe in die hede te bevorder nie, maar ook dien as ’n meganisme waardeur tradisionele kennis van rampgebeure van geslag tot geslag oorgedra word. Vervolgens word ’n diepgaande korpus- en n-gram-analise van rampverwante terminologie in Afrikaans uitgevoer. Die artikel sluit af met insigte oor wat hierdie terminologie ons vertel oor Afrikaanssprekendes se begrip en perspektief van rampe.

## Die ontwikkeling van Afrikaans as wetenskapstaal

Volgens Carstens (aangehaal deur Van Oort 2008:74), stel kennis van die historiese verlede van ’n taal ’n mens in staat om ’n beter begrip en perspektief op die hede en die toekoms van daardie taal te verkry. Die historiese ontwikkeling van Afrikaans is kompleks en is reeds deur verskeie taalteoretici gedokumenteer en gedebatteer. Van Oort (2008) beklemtoon hierdie kompleksiteit in die historiese ontwikkeling van Afrikaans deur 40 taalteoretici se perspektiewe oor die ontstaan en ontwikkeling van die taal, asook die invloed van sosiopolitieke faktore, te bestudeer. Volgens Van Oort (2008) kan die ontwikkelingsgeskiedenis van Afrikaans hoofsaaklik in twee tydvlakke verdeel word (1652–1799 en 1800–2000) waartydens drie variëteite ontstaan het, naamlik die historiese Westelike, Suidelike en Oostelike variëteite van Afrikaans.

Die ideologiese diskoerse waaruit linguistiese purisme in akademiese tekste wat handel oor die ontwikkeling van Afrikaans voortgespruit het, word verder deur Kotze en Kirsten (2016) bevraagteken. Alhoewel sommige ontwikkelingsperspektiewe ooreenstem, bestaan daar steeds talle meningsverskille. Ten spyte van ’n komplekse historiese ontwikkeling is Afrikaans in 1925 as amptelike taal in die Unie van Suid-Afrika erken. Later, in 1996, is Afrikaans weereens as een van die amptelike tale van Suid-Afrika in die Republiek se Grondwet bevestig (Hoofstuk 1 (6)(1)) (Republiek van Suid-Afrika 1996:4).

Na die implementering van die Grondwet is daar toenemend klem geplaas op die ontwikkeling van die land se amptelike tale, insluitend Afrikaans, met ’n spesifieke fokus op die bevordering van Afrikaans as wetenskaps- of vaktaal. Alberts (2016:318) beskryf vaktaal as die ‘basis van spesialiskommunikasie’ wat die ‘skep, ontsluiting en verspreiding van kennis en inligting’ fasiliteer. Die historiese ontwikkeling van Afrikaans as wetenskapstaal was egter ’n≈moeisame ontwikkelingsproses (Alberts 2016, 2017). Wolfaardt-Gräbe (2015:511) identifiseer drie spesifieke probleme wat die bevordering van Afrikaans as wetenskapstaal belemmer: gebrekkige direkte vertaling uit Engels; die ‘verarming van Afrikaans se seggingskrag’; en inkonsekwentheid in skryfstyle en terminologiegebruik. Alhoewel elke spesialisveld sy eie gestandaardiseerde terminologie ontwikkel, bly voortdurende vooruitgang in die skepping en begrip van terme noodsaaklik vir effektiewe kennisverwerwing en -oordrag (Alberts 2016, 2017). Vir die suksesvolle en doeltreffende oordrag van vak- of beroepsgerigte inligting en kennis is ondubbelsinnige, sinvolle kommunikasie noodsaaklik vir die toekomstige ontwikkeling van Afrikaans as wetenskapstaal. Die volgende afdeling fokus gevolglik op die belangrikheid van Afrikaans as moedertaal, veral in die konteks van rampverstaan en -vermindering.

## Die belangrikheid van moedertaal in die verstaan en vermindering van rampe

Volgens Alexander en Pescaroli (2019:2) en Zhang et al. (2019:190–191) het baie van die teoretiese en praktiese ontwikkelings met betrekking tot rampkommunikasie oor die afgelope paar dekades grootliks gefokus op tegniese meganismes, soos vroeë waarskuwingstelsels en die verhoogde gebruik van sosiale media, om bewustheid en begrip van rampe te bevorder. Hulle redeneer egter dat, ten spyte van hierdie kontemporêre fokus op die meganismes van rampkommunikasie, die gebruik van ’n gemeenskap se moedertaal (bv. Afrikaans) steeds ’n fundamentele rol speel in die begrip van rampe en die impak daarvan op ’n gemeenskap.

’n Vlugtige oorsig van die literatuur dui daarop dat ’n gemeenskap se moedertaal verskeie funksies vervul in die vermindering van ramprisiko. Die primêre rol van moedertaal hou verband met sy vermoë om begrip te skep van ’n spesifieke gebeurtenis, konsep of onderwerp. In die geval van rampe, dien moedertaal as ’n meganisme waardeur ’n gemeenskap oor rampgebeure kan reflekteer en aan die sosiale konstruksie van hulle ervaring van rampe kan deelneem. Deur hierdie proses word ’n unieke kulturele lens en raamwerk geskep wat die gemeenskap se toekomstige interaksie met rampe rig (Breidlid 2009:141; Lunga & Musarurwa 2016:23). Magni (2017:438–439) verduidelik dat moedertaal kultuurspesifieke ervarings, mites, oortuigings en inheemse kennissisteme ontsluit oor toepaslike strategieë om toekomstige rampgebeure te hanteer en die impak daarvan te verminder. Berkes et al. (2021:7) beklemtoon die waardevolle rol van moedertaal in die verstaan en bestuur van ekologiese prosesse (bv. rampe) in hulle gevallestudie oor Taiwan. Hulle beskryf dat daar tans 16 inheemse groepe op die eiland woon en dat elkeen hulle unieke taal- en kulturele begrippe ontwikkel het vir verskeie omgewingselemente (bv. biodiversiteit en rampe) wat die eiland oor eeue heen beïnvloed het. Hierdie ryke taalbeskrywings het bygedra tot die vorming van ’n unieke kennisbasis wat inheemse gemeenskappe hoogs aanpasbaar en veerkragtig maak teen rampgebeure. Daar word verder aanbeveel dat hierdie tradisionele kennis benut moet word om kontemporêre benaderings tot ramprisikovermindering (DRR) op die eiland te verbeter. Inheemse kennisstelsels, soos hierbo beskryf, is gegrond op moedertaaltaalgebruik en bied ’n unieke benadering tot ramprisikovermindering, onafhanklik van Eurosentriese modelle wat dikwels nie voldoende aangepas is vir die Afrika-konteks nie (Chmutina & Von Meding 2019:290; Howitt, Havnen & Veland 2012:53).

Tweedens dien ’n gemeenskap se moedertaal as ’n meganisme vir die oordrag van tradisionele kennis van een geslag na die volgende. Breidlid (2009:142) en Cuaton en Su (2020:23) stem saam dat kennis oor vorige rampgebeure en omgewingsbestuur deur mondelinge tradisies (bv. stories en mitologie) oorgedra word om vaardigheidsontwikkeling en ramphanteringskapasiteit tussen generasies te bevorder. Ali et al. (2021:10) bevestig hierdie waarneming van Breidlidin in hulle werk met die Yolηu-volk in Galiwin’ku, Noord-Australië. Hulle het bevind dat die oudstes binne die gemeenskap inligting in hulle moedertaal aan die jeug oordra oor natuurverskynsels, soos die sewe verskillende seisoene binne ’n jaar en die gepaardgaande plant- en dieregedrag wat as vroeë waarskuwingstekens dien vir moontlike sikloonvorming. Hierdie geval illustreer hoe moedertaal ’n fundamentele rol speel om te verseker dat kennis oor vorige rampe en die vaardighede om hierdie impakte te hanteer, van geslag tot geslag oorgedra word. Lunga en Musarurwa (2016:25) en Ramadhan, Sukma en Indriyani (2019:2) beklemtoon dat daar ’n behoefte is aan die ontwikkeling van ’n wetenskaplike taal en terminologie in inheemse tale (d.w.s. in persone se eie moedertaal) om te verseker dat inheemse gemeenskappe hulle waardevolle kennis en unieke benadering tot rampbestuur integreer in die vakgebied van Rampriskobestuur. Lambert en Scott (2019:11) voeg by dat die ontwikkeling van terminologie in inheemse tale kan help om te verhoed dat sekere tale uitsterf en sodoende waardevolle inheemse kennis, insluitend dié oor rampe, te bewaar.

Laastens speel moedertaal ’n sleutelrol in die bevordering van gemeenskapsbetrokkenheid by ramprisikovermindering-intervensies (Mercer et al. 2008:177). In hulle studie onder die Anishinabe-nasie in Manitoba, Kanada, bevind Buckland en Rahman (1999:188) dat die gebruik van hulle inheemse taal, Ojibway, sosiale kapitaal ontwikkel het wat verseker het dat ontwikkelingsaktiwiteite (bv. rampsassesserings) volhoubaar sowel as deelnemend van aard was. Mecer et al. (2008:173–174) argumenteer dat ramprisikobestuursaktiwiteite in die verlede dikwels verkeerd benader is, aangesien gemeenskappe as passiewe ontvangers van ramprisikostrategieë beskou is en nooit ten volle kon deelneem aan die formulering van strategieë om hulle eie ramprisiko te verminder nie. Deur benaderings soos deelnemende aksie-navorsing en om deelnemende leer en aksie (PLA) te integreer in die formulering van ramprisikobestuursaktiwiteite, en hierdie aktiwiteite in die moedertaal van deelnemers uit te voer, word hierdie prosesse meer toeganklik. Dit stel gemeenskappe in staat om hulle begrip van ramprisiko en moontlike oplossings in hulle eie taal uit te druk. Weichselgartner en Pigeon (2015:110), en Cuaton en Su (2020:5) voeg by dat wanneer gemeenskappe toegelaat word om hulleself in hulle moedertaal uit te druk, nuwe en ramprisikostrategieë dikwels na vore kom, wat voorheen deur buitestanders misgekyk is. Verder sluit die unieke benaderings aan by die konteks waarin die gemeenskap hulle bevind, en dit dra by tot relevansie en volhoubaarheid van voorgestelde oplossings.

Volgens Magni (2017:440) kan die belangrikheid van moedertaal in die ontwikkeling van ramprisikovermindering nie oorbeklemtoon word nie. Wanneer ’n gemeenskap hulleself nie in hulle eie taal kan uitdruk nie, is hulle minder geneig om aan ontwikkelingsprogramme deel te neem en hulle inheemse kennis en ervaring tot die volhoubaarheid van hierdie programme by te dra. Vanweë die beduidende bydrae van moedertaal in die bevordering van kultuurspesifieke begrip en reaksies op rampe, is dit uiters belangrik om die oorsprong van sekere rampverwante woordeskat en konsepte binne ’n taal te verstaan. Verder is dit ook noodsaaklik om te verstaan hoe hierdie terminologie oor tyd binne ’n spesifieke kultuur ontwikkel en geïnterpreteer is (Alexander & Pescaroli 2019:4). Die volgende afdeling fokus gevolglik op die etimologie van die konsep ‘ramp’ in die Afrikaanse taal en wat dit oor Afrikaanssprekendes se verstaan van hierdie konsep onthul, na aanleiding van die ontwikkeling van die woord in die taal. Voordat hierdie analise onderneem word, word die navorsingsmetodologie vir die studie eers uiteengesit.

## Metodologie

Hierdie studie volg ’n kwalitatiewe navorsingsbenadering wat, volgens Fouché en Delport (2011), geskik is vir studies wat die aard van ’n navorsingsprobleem of verskynsel ondersoek en daarop gefokus is om die probleem te beskryf en te verstaan, eerder as om menslike gedrag te verduidelik of te voorspel. Dokumentanalise is as kwalitatiewe datainsamelingstegniek gebruik. Volgens Fouche en Delport (2011) behels dokumentanalise die studie van bestaande dokumente om ‘n dieper betekenisse daaruit te ontgin.

Korpusanalise is toegepas om die begrip van die konsep ‘ramp’ in Afrikaans oor die algemeen te bestudeer, met spesifieke fokus op die wyse waarop dit in verskillende kontekste gebruik word. Die omvattende korpusversameling op VivA se Korpusportaal (VivA 2022) is gebruik as ’n gepaste datastel, aangesien dit ’n wye reeks Afrikaanse taalgebruik uit verskillende bronne verteenwoordig.

Hierdie omvattende korpusversameling bestaan tans uit byna 300 miljoen woorde, wat uit 11 sub-korpora saamgestel is. Die verteenwoordigende genres sluit onder meer regeringsdokumente, toneelstukke, fiksie en nie-fiksie van verskeie uitgewers, aanlyn-nuusartikels, radio-nuusbulletins, spontane gesproke taal, die Afrikaanse Wikipedia en die Taalkommissie-korpus in. Laasgenoemde bestaan uit verskeie tekstipes, insluitend akademiese tekste, koerante, fiksie en ander skryfstukke.

Die Korpusportaal-platform bied verskeie metodes om data te onttrek en berekenings te maak. Die eerste datastel wat vir hierdie studie onttrek is, behels die gebruiksgevalle van die woord ‘ramp’, sowel as alle samestellings wat met ‘ramp’ begin of met ‘ramp’ eindig. Dieselfde prosedure is daarna toegepas op twee ander terme, ‘katastrofe’ en ‘krisis’, wat in gebruik en betekenis met ‘ramp’ oorvleuel. Deur te ondersoek watter konsepte dikwels in kombinasie met hierdie terme voorkom, kan afleidings gemaak word oor die aspekte van die verskynsels waarop taalgebruikers dikwels fokus.

’n Verdere analise is met behulp van die Korpusportaal-platform uitgevoer, naamlik die ontleding van n-gramme. Hierdie ontleding bepaal watter spesifieke woorde of woordkombinasies vóór of ná die soekwoord gebruik word en hoe gereeld dit saam voorkom. Vir hierdie studie is die volgende parameters gebruik: een woord vóór ‘ramp’; twee woorde vóór ‘ramp’; een woord ná ‘ramp’; en twee woorde ná ‘ramp’. Die afsnypunt was telkens dat die betrokke woordkombinasies minstens 10 keer in die korpusdata moes voorkom. Die twee stelle n-gramme van woorde wat ‘ramp’ voorafgaan, is daarna saamgevoeg om vas te stel watter woorde en kombinasies dit gereeld voorafgaan. Dieselfde metode is toegepas op woorde wat op ‘ramp’ volg. Hierdie proses is telkens herhaal vir ‘katastrofe’ en ‘krisis’.

Die n-gram-analise verskaf meer inligting oor die kontekste waarin hierdie terme gebruik word, wat help om die belangrikste gebruikskontekste van ‘ramp’ vas te stel. Dit bied ook ’n vergelykende perspektief op die ooreenkomste en verskille in die kontekste waarbinne ‘katastrofe’ en ‘krisis’ gebruik word.

Die volgende afdeling bied ’n verdere oorsig van die bevindings oor die etimologie van die woord ‘ramp’ in Afrikaans.

### Etiese oorwegings

Hierdie artikel het alle etiese standaarde gevolg vir navorsing sonder direkte kontak met, en deelname van mense.

## Die etimologie van die woord ‘ramp’ in Afrikaans

Volgens Van der Sijs (2010) het die woord ‘ramp’ in Afrikaans sy oorsprong in Middelnederlands, waar dit ook as ‘rampe’ en ‘rampa’ voorgekom het, met ’n algemene betekenis van ellende of onheil. Die basis van die woord is die Germaanse ‘rimp’ of ‘χrimp’, wat ‘om saam te trek’ beteken en waaruit woorde soos ‘rimpel’ ook ontwikkel het (Van der Sijs 2010). Philippa et al. (2007) dui aan dat die woord ‘ramp’ aan ‘rimpel’ verwant is, soos ‘kramp’ aan ‘krimp’ verwant is. Verder het ‘ramp’ in Middelnederlands ook die betekenis van ‘vallende siekte’ of ‘kramp’ gehad. Van Wyk (red. 2007) verduidelik dat die woord ‘ramp’ in Afrikaans betekenisse soos ‘onheil’ en ‘slegte ervaring’ het. ’n Belangrike observasie is dat die woord ‘ramp’ in Afrikaans ’n Germaanse oorsprong het en dat daar min invloed vanuit ander inheemse tale op die ontwikkeling van die woord is – anders as wat dikwels die geval is met baie ander Afrikaanse woorde.

Volgens Botha (red. 2009:41) word ‘ramp’ in Deel XIII van die *Woordeboek van die Afrikaanse Taal* (WAT) gedefinieer as ‘’n oorweldigende, dikwels onvoorsiene gebeurtenis of voorval wat gewoonlik gepaardgaan met groot ellende of teenspoed, of grootskaalse skade, vernietiging of verlies’. Die *Wet op Rampbestuur* (57/2002) bou hierop voort en definieer ’n ramp as:

’n Progressiewe of skielike, wydverspreide of gelokaliseerde, natuurlike of mensgemaakte gebeurtenis wat (a) (i) dood, besering of siekte; (ii) skade aan eiendom, infrastruktuur of die omgewing; of (iii) ontwrigting van ’n gemeenskap veroorsaak of dreig om te veroorsaak; en (b) van ’n omvang is wat die vermoë oorskry van diegene wat deur die ramp geraak word om sy gevolge die hoof te bied deur slegs van hul eie hulpbronne gebruik te maak. (Republik van Suid Afrika, 2002:7)

Met betrekking tot sinonieme is daar ’n verskeidenheid woorde in *Sinonieme en verwante woorde* (Harteveld 2006:310) opgeneem te wete katastrofe, kalamiteit, nekslag, ernstige ongeluk, groot ongeluk, natuurramp, rampspoed, ellende, groot nood, ongeluk, teenspoed, onheil en nekslag. Van hierdie woorde is ‘katastrofe’ die sinoniem wat die nouste ooreenstem met ‘ramp’ in terme van woordeboekbetekenis. In Deel V van die WAT word ‘katastrofe’ in sy tweede betekenis omskryf as:

’n Groot, oorweldigende gebeurtenis wat plotseling, soms na ’n voorbereidende aanloop, as ’n uiters ongelukkige, ontwrigtende of rampspoedige, vernietigende ommekeer of omwenteling in ’n lewensamehang of lewensverloop … in die natuur of van ’n persoon plaasvind. (red. Snijman 1968:405)

Hoewel ‘krisis’ nie as sinoniem vir ‘ramp’ in die lys opgeneem is nie, word ‘ramp’ wel as sinoniem vir ‘krisis’ aangedui (Harteveld 2006:202). Wanneer die omskrywing van ‘krisis’ bestudeer word, is dit duidelik dat daar ’n verwantskap tussen die twee begrippe bestaan. Hauptfleisch (1991) definieer ‘krisis’ in Deel VIII van die WAT as:

’n Beslissende of betekenisvolle, ingrypende tydstip of stadium in die verloop van sake of gebeure, of in die gesteldheid of bestaan van ’n persoon of groep, wanneer ’n hoogtepunt of keerpunt bereik word, gewoonlik met ’n gunstige of ongunstige beïnvloeding van die verdere verloop van die toestand. (p. 201)

Luther, Pheiffer en Gouws (2015:689) se definisie in die HAT is nader aan die manier waarop ‘krisis’ in die algemeen in Afrikaans gebruik word: ‘Situasie waarin daar ’n ernstige probleem is wat vinnig hanteer/opgelos moet word, sodat die toestand nie verder versleg nie.’ Ander woorde soos ‘natuurramp’, ‘kalamiteit’, ‘rampspoed’, ‘gatslag’, ‘onheil’, ‘nekslag’, ‘ongeluk’, ‘jammer’, ‘mislukking’ en ‘slag’ word ook soms as sinonieme vir ‘ramp’ beskou, maar is volgens hulle gebruiksvoorkoms-omskrywing, of omdat ‘ramp’ ’n onderdeel van die woord is, as sinonieme tydens die skryf van hierdie manuskrip geïdentifiseer.

## Woorde wat saam met ‘ramp’, ‘krisis’ en ‘katastrofe’ gebruik word

Daar is veral twee soorte woorde wat in gereelde gebruikspatrone die woord ‘ramp’ met een woord voorafgaan in die korpusse wat ontleed is, naamlik beskrywende woorde en woorde wat die tipe ramp aandui (Tabel 1). Rampe word in die meeste gevalle as ‘nasionaal’ of ‘groot’ beskryf, terwyl beskrywings soos ‘verskriklik’ en ‘tragies’ as sinonieme vir ‘groot’ kan dien. Van die tipes rampe wat genoem word, kom ‘ekonomies’ en ‘finansieel’ die meeste voor, gevolg deur ‘humanitêr’, ‘menslik’ en ‘mensgemaakte’. Daarna volg ‘ekologiese’ rampe, terwyl ‘natuurlike’ rampe die kleinste kategorie vorm. Wanneer twee woorde voorafgegaan word, blyk konsepte soos hoeveelheid (‘oormaat’), tyd (‘tydens’) en gebeurtenis (‘tref’) prominent te wees.

**TABEL 1:** Woorde wat gereeld ‘ramp’, ‘katastrofe’ en ‘krisis’ voorafgaan.

Aard van woord	Woord	Ramp	Katastrofe	Krisis
Beskrywend	absolute	x		
	ander	x		
	daaglikse		x	
	dreigende	x	x	x
	enorme		x	x
	erge			x
	ernstige			x
	geweldige			x
	globale		x	
	groeiende			x
	groot	x	x	x
	huidige			x
	internasionale			x
	jong	x		x
	klein		x	
	komende			x
	moontlike	x		x
	nasionale	x		x
	nuwe			x
	ongekende			x
	potensiële	x	x	
	soortgelyke	x		
	toenemende			x
	totale	x		
	tragiese	x		
	verskriklike	x		
	voortdurende			x
	voortslepende			x
	wêreldwye		x	x
	werklike			x
Tipe	(plek)			x
	boere			x
	demografiese			x
	diplomatiese			x
	ekologiese	x	x	x
	ekonomiese	x	x	x
	eksistensiële			x
	emosionele			x
	finansiële	x		x
	fiskale			x
	geestelike			x
	geldelike			x
	grondwetlike			x
	humanitêre	x	x	x
	konstitusionele			x
	maatskaplike			x
	mediese			x
	mensgemaakte	x		
	menslike	x		
	morele			x
	natuurlike	x		
	persoonlike			x
	politiese			x

Hoewel die omskrywing van ‘katastrofe’ nader aan dié van ‘ramp’ as dié van ‘krisis’ is, kom daar beduidend meer verwysings na ‘krisis’ in die korpusse voor. Daar is ’n groot aantal tipes krisisse wat beskryf word, waarvan ‘ekonomies’, ‘finansieel’, ‘polities’, ‘humanitêr’ en ‘ekologies’ die meerderheid uitmaak. Voorts word ‘krisis’ beskryf in terme van tyd (‘huidig’, ‘voortslepend’, ‘nuut’, ‘jonk’, ‘voortdurend’), plek en omvang (‘groot’, ‘nasionaal’, ‘wêreldwyd’, ‘internasionaal’, ‘enorm’, ‘ongekend’, ‘geweldig’), aard (‘ernstig’, ‘dreigend’, ‘toenemend’, ‘erg’, ‘werklik’) en potensiaal (‘groeiend’, ‘moontlik’, ‘komend’). In die korpusse word ‘katastrofe’ meestal beskryf in terme van grootte of omvang (‘groot’, ‘wêreldwyd’, ‘globaal’, ‘klein’, ‘enorm’), frekwensie (‘daagliks’) en potensiaal (‘dreigend’, ‘potensieel’). Die tipes katastrofe waarna verwys word, sluit ‘humanitêr’, ‘ekonomies’ en ‘ekologies’ in.

Met betrekking tot woorde wat onmiddellik op ‘ramp’ volg in die korpusse wat gebruik is, is daar drie hoofsoorte: (1) dinge wat *gedoen* word; (2) dinge wat *ervaar* word; en (3) dinge wat *gebeur* (Tabel 2). Dinge wat gedoen word in terme van rampe, sluit woorde in soos ‘verklaar’, ‘beskou’, ‘beskryf’, ‘veroorsaak’, ‘bestempel’ en ‘afweer’, terwyl ‘voorkom’ en ‘verhoed’ bykom wanneer twee woorde ná ‘ramp’ in ag geneem word. Die woorde ‘afweer’, ‘voorkom’ en ‘verhoed’ dui op ’n moontlike begrip van ramprisikovermindering.

**TABEL 2:** Woorde wat gereeld op ‘ramp’, ‘katastrofe’ en ‘krisis’ volg.

Aard van woord	Woord	Ramp	Katastrofe	Krisis
Doen	afweer	x		
	beheer			x
	beskou	x		x
	beskryf	x		x
	bespreek			x
	bestempel	x		x
	hanteer			x
	help			x
	oplos			x
	probeer			x
	skep			x
	vererger			x
	verklaar	x		x
	vermy		x	
	veroorsaak	x		x
	vind			x
	voorkom			x
Ervaar	beland			x
	beleef		x	x
	bevind			x
	dood	x		
	ervaar			x
	omkom	x		
	oorleef	x		x
	raak			x
	sterf	x		
	tref	x		
	verkeer			x
	verloor	x		
Gebeur	afstuur	x		x
	begin			x
	dompel			x
	dreig			x
	gebeur	x		
	geëindig	x		
	lei	x		x
	ontaard	x	x	x
	ontstaan			x
	opduik			x
	plaasvind	x		
	tot gevolg		x	
	uitloop	x		

Die meeste woorde wat met ervaring verband hou, verwys na lewe en dood, byvoorbeeld ‘oorleef’, ‘dood’, ‘omkom’ en ‘sterf’. Verder word mense ook deur rampe ‘getref’, of hulle verloor hulle lewe, lewensbestaan of besittings.

Gebeure in verband met rampe sluit woorde in soos ‘afstuur’, ‘plaasvind’, ‘uitloop’, ‘ontaard’, ‘lei’, ‘gebeur’ en ‘geëindig’. Hierdie woorde hou oorwegend verband met die oorsaak en gevolg, of die ontwikkeling van ’n ramp. Laastens is daar een beskrywende woord, ‘verantwoordelik’, wat ook met oorsaak en gevolg verband hou. Interessant genoeg, hoewel die omskrywing van katastrofe meer met ‘ramp’ as ‘krisis’ ooreenstem, is daar beduidend meer verwysings in die korpusse na ‘krisis’ as na ‘katastrofe’. Krisisse word soos ‘katastrofe’ en ‘ramp’ ervaar, maar die menslike aspek is meer prominent, byvoorbeeld woorde soos ‘beland’, ‘beleef’, ‘ervaar’ en ‘oorleef’. Beskrywings van krisisse met woorde wat volg, fokus op plek en tyd met die spesifieke verwysings na jaartal of land/instelling. Gebeure rondom ‘krisis’ oorvleuel in ’n groot mate met ‘ramp’, maar die sterk assosiasie met ‘dompel’ is uniek tot ’n krisis. Aksies met betrekking tot ‘krisis’ stem insgelyks in ’n groot mate met ‘ramp’ ooreen, maar ook hier is die menslike aspek sigbaar in terme soos ‘vererger’, ‘oplos’, ‘skep’ en ‘beheer’. Die woorde wat op ‘katastrofe’ volg, hou verband met ervaring (‘beleef’), gebeure (‘ontaard’, ‘tot gevolg’) en aksie (‘voorkom’, ‘vermy’). Hierdie terminologie dui ook op ’n begrip van ramprisikovermindering binne die konteks van taalgebruik.

## Woorde in samestellings met ‘ramp’, ‘krisis’ en ‘katastrofe’

Hierdie afdeling bied ’n beskrywing van die samestellings waarin die woorde ‘ramp’, ‘krisis’ en ‘katastrofe’ voorkom. In plaas daarvan om op gebruiksfrekwensies te fokus, word daar aandag geskenk aan woorde wat in die meeste verskillende samestellings gebruik word. Dit gee ’n aanduiding van die spesifieke kontekste waarin verskillende rampe, krisisse en katastrofes voorkom.

Daar is 796 samestellings met ‘ramp’ uit die korpusdata onttrek, waarvan 450 met ‘ramp’ begin en 346 met ‘ramp’ eindig. Die woord wat die meeste saam met ‘ramp’ in verskeie samestellings voorkom, is ‘bestuur’, met voorbeelde soos ‘rampbestuuradviesforum’, ‘rampbestuurkonferensie’, ‘rampbestuursafdeling’, ‘rampbestuurwoordvoerder’ en die algemene term ‘rampbestuur’. Ander woorde wat dikwels in kombinasie met ‘ramp’ gebruik word, sluit in ‘hulp’ (bv. ‘ramphulpfonds’, ‘ramphulpskema’), ‘verligting’ (bv. ‘rampverligtingsprojek’, ‘rampverligting-organisasie’), en ‘risiko’ (bv. ‘ramprisikobestuur’, ‘ramprisikostudies’). Wat samestellings betref wat met ‘ramp’ eindig, sluit ’n groot aantal hiervan eiename, soos ‘Driehoekramp’, ‘Septemberramp’ en ‘Zuma-ramp’ in. Die tipes rampe waarvan daar baie verskillende gevalle in die data voorkom, sluit veral busrampe, kernrampe, mynrampe, en lug- en vliegrampe in.

Die woord ‘katastrofe’ kom in relatief min samestellings voor – slegs 45 samestellings is uit die data onttrek. Hiervan begin slegs ses met ‘katastrofe’, terwyl 39 met ‘katastrofe’ eindig. Daar is geen woorde wat in meer as een samestelling met ‘katastrofe’ voorkom nie.

In teenstelling hiermee word ‘krisis’ gereeld in samestellings gebruik, met ’n totaal van 880 samestellings wat uit die data onttrek is. Hiervan begin 178 met ‘krisis’, terwyl 702 met ‘krisis’ eindig. Soos met ‘ramp’ word ‘krisis’ ook dikwels in samestellings met ‘bestuur’ gebruik, hoewel dit nie so prominent soos by ‘ramp’ is nie. Die meer prominente kombinasies kom voor in samestellings wat met ‘krisis’ eindig, en die tipe rampe wat die meeste voorkom, sluit in kragkrisisse (saam met elektrisiteitskrisisse en energiekrisisse), skuldkrisisse, voedselkrisisse, waterkrisisse en wêreldkrisisse.

Uit bogenoemde ontleding kan afgelei word dat die fokus, wanneer Afrikaanssprekendes van rampe praat, effens verskil van wanneer hulle van krisisse praat. Rampe verwys tipies na ’n spesifieke gebeurtenis – dikwels ’n ongeluk van een of ander soort, of natuurverskynsels. Krisisse, daarenteen, verwys eerder na langdurige toestande wat dikwels deur menslike optrede veroorsaak word, soos elektrisiteitstekorte, watertekorte, skuldprobleme, en so meer. Die drie terme waarop gefokus word in hierdie studie kom ook tog in soortgelyke samestellings voor:

- ramp/katastrofe/krisisgebied
- brandramp/katastrofe/krisis
- gesondheidsramp/katastrofe/krisis
- kernramp/katastrofe/krisis
- klimaatsramp/katastrofe/krisis
- landbouramp/katastrofe/krisis
- natuurramp/katastrofe/krisis
- olieramp/katastrofe/krisis
- omgewingsramp/katastrofe/krisis
- reuseramp/katastrofe/krisis
- wêreldramp/katastrofe/krisis

Hierdie oorvleuelende samestelling toon aan dat die kernbetekenis waarna ‘ramp’ kan verwys inderdaad met ‘katastrofe’ en ‘krisis’ ooreenstem, alhoewel daar duidelike verskille in die tipiese gebruikspatrone van elke term is.

## Slotsom

Opsommend blyk dit uit die data dat die woorde wat ‘ramp’, ‘katastrofe’ en ‘krisis’ voorafgaan in twee groepe verdeel kan word: *beskrywend* en *tipe*. Dit is interessant om te let op die gebruik van ‘menslik’, ‘mensgemaak’ en ‘natuurlik’ wat slegs ‘ramp’ voorafgaan, wat met die definisie van ‘ramp’, soos in die *Wet op Rampbestuur* (57/2002) vervat, ooreenstem. In die akademiese gemeenskap word daar egter algemeen aanvaar dat rampe nié as natuurlike verskynsels beskou moet word nie, maar dat daar wel natuurgevare is wat tot rampe kan lei; dus is gevare natuurlik, maar rampe nie. Die korpusse weerspieël dus eerder die wetgewing as die siening van die Verenigde Nasies se Kantoor vir Ramprisikovermindering (UNDRR) (UNISDR 2009), wat ’n duidelike onderskeid tref tussen natuurgevare en rampe. Verder is dit interessant dat ‘humanitêr’ al drie trefwoorde voorafgaan, terwyl die definisie in die Wet sowel as die UNDRR daarop dui dat rampe slegs op die mens betrekking het, wat beteken dat die gebruik van ‘humanitêr’ in hierdie konteks oorbodig kan wees.

Woorde wat op ramprisikovermindering betrekking het en wat op die drie trefwoorde volg, lewer ook insiggewende resultate op. In terme van *doen*-woorde wat volg, is dit terme soos ‘hanteer’, ‘help’, ‘oplos’, ‘probeer’ en ‘voorkom’ wat slegs op ‘krisis’ volg, terwyl ‘vermy’ slegs op ‘katastrofe’ volg. Daar is egter geen van die *doen*-woorde wat op ‘ramp’ volg, wat dan op die verminderingsaspek van ramprisikovermindering dui nie. Hieruit blyk dit dat die gebruik van die woord ‘ramp’ in die korpusse nie tred hou met die mees onlangse neigings in die vakgebied nie; met ander woorde, dat rampe voorkombaar is en dat proaktiewe aksies geneem moet word om die impak daarvan op die samelewing te verminder. Dit is daarom van kardinale belang om die gebruik van die woord ‘ramp’ binne die Afrikaanse taal aan te pas deur die *doen*-woorde van konsepte soos ‘krisis’ en ‘katastrofe’ te inkorporeer, aangesien die *doen*-woorde ooreenstem met die proaktiewe bestuur van rampe, soos deur globale sowel as nasionale rampbestuurwetgewing aangemoedig word.

Verder is dit ook belangrik om die gebruik van die konsep van rampriskobestuur meer gereeld te inkorporeer in gesprekke oor rampe in die Afrikaanse media en skoolkurrikula. Dit kan help om ’n kulturele verskuiwing in denke teweeg te bring, waarin die besef groei dat rampe nie noodwendig buite ons beheer is nie en dat proaktiewe ramprisko-verminderingstrategieë wel beskikbaar is om die impak daarvan te verminder of selfs te voorkom.

Wat samestellings betref, toon die data ’n soortgelyke patroon: ‘krisis’ word in meer samestellings gebruik as ‘ramp’, terwyl ‘katastrofe’ beduidend minder in samestellings voorkom. ’n Opvallende verskil tussen ‘ramp’ en ‘krisis’ in samestellings is dat ‘ramp’ hoofsaaklik na spesifieke gebeurtenisse verwys, terwyl ‘krisis’ gebruik word in die konteks van mensgemaakte gebeure of deur menslike toedoen. Dit is veelseggend dat die voorvoegsels ‘natuur-’ en ‘omgewing-’ prominent by samestellings figureer deurdat dit met al drie die terme gekombineer word. Dit dui op ’n konseptualisering wat afwyk van die akademiese siening dat natuurlike gebeurtenisse as sodanig nie rampe is nie. Hierdie konseptualisering suggereer ’n meer reaktiewe ingesteldheid, wat impliseer dat rampe onvermydelik is. Indien die samelewing, en in hierdie geval spesifiek die Afrikaanse spraakgemeenskap se konseptualisering van ‘rampe’ aangepas kan word om ’n meer proaktiewe benadering te weerspieël, kan dit ’n positiewe impak hê op ramprisikovermindering.

Afrikaans as moedertaal speel ’n fundamentele rol in die skepping en verstaan van konsepte, asook in die ontwikkeling van strategieë vir ramprisikovermindering. Dit is dus van kardinale belang dat Afrikaanssprekendes aangemoedig word om meer aktief betrokke te raak by rampbestuursaktiwiteite en om volhoubare oplossings tydens rampe te ontwikkel. Die vermoë om hierdie konsepte in hulle moedertaal uit te druk, kan verder bydra tot ’n beter begrip van en reaksie op rampe binne Afrikaanse gemeenskappe.
